# A modular plasmid toolkit applied in marine bacteria reveals functional insights during bacteria-stimulated metamorphosis

**DOI:** 10.1128/mbio.01502-23

**Published:** 2023-08-02

**Authors:** Amanda T. Alker, Morgan V. Farrell, Alpher E. Aspiras, Tiffany L. Dunbar, Andriy Fedoriouk, Jeffrey E. Jones, Sama R. Mikhail, Gabriella Y. Salcedo, Bradley S. Moore, Nicholas J. Shikuma

**Affiliations:** 1 Department of Biology, San Diego State University, San Diego, California, USA; 2 Center for Marine Biotechnology and Biomedicine, Scripps Institution of Oceanography, University of California, San Diego, California, USA; University of Hawaii at Manoa, Honolulu, Hawaii, USA

**Keywords:** CRISPRi, golden gate, violacein, metamorphosis, tubeworm, *Hydroides*, modular, marine, symbiosis

## Abstract

A conspicuous roadblock to studying marine bacteria for fundamental research and biotechnology is a lack of modular synthetic biology tools for their genetic manipulation. Here, we applied, and generated new parts for, a modular plasmid toolkit to study marine bacteria in the context of symbioses and host-microbe interactions. To demonstrate the utility of this plasmid system, we genetically manipulated the marine bacterium *Pseudoalteromonas luteoviolacea*, which stimulates the metamorphosis of the model tubeworm, *Hydroides elegans*. Using these tools, we quantified constitutive and native promoter expression, developed reporter strains that enable the imaging of host-bacteria interactions, and used CRISPR interference (CRISPRi) to knock down a secondary metabolite and a host-associated gene. We demonstrate the broader utility of this modular system for testing the genetic tractability of marine bacteria that are known to be associated with diverse host-microbe symbioses. These efforts resulted in the successful conjugation of 12 marine strains from the Alphaproteobacteria and Gammaproteobacteria classes. Altogether, the present study demonstrates how synthetic biology strategies enable the investigation of marine microbes and marine host-microbe symbioses with potential implications for environmental restoration and biotechnology.

**IMPORTANCE:**

Marine Proteobacteria are attractive targets for genetic engineering due to their ability to produce a diversity of bioactive metabolites and their involvement in host-microbe symbioses. Modular cloning toolkits have become a standard for engineering model microbes, such as *Escherichia coli*, because they enable innumerable mix-and-match DNA assembly and engineering options. However, such modular tools have not yet been applied to most marine bacterial species. In this work, we adapt a modular plasmid toolkit for use in a set of 12 marine bacteria from the Gammaproteobacteria and Alphaproteobacteria classes. We demonstrate the utility of this genetic toolkit by engineering a marine *Pseudoalteromonas* bacterium to study their association with its host animal *Hydroides elegans*. This work provides a proof of concept that modular genetic tools can be applied to diverse marine bacteria to address basic science questions and for biotechnology innovations.

## INTRODUCTION

Marine bacteria are a valuable and currently under-utilized resource for environmental restoration (1 - 6) and bioprospecting (7, 8), especially considering their influence on biogeochemical cycles (9) and their vital role in evolution through symbioses with eukaryotes (10). While advances in metagenomic sequencing have enabled a deeper exploration of microbial diversity and gene content (11, 12), genetic tools to explore functions in marine bacteria remain scarce.

Effective genetic engineering approaches in model microbial species, such as *Escherichia coli*, utilize standardized and modular cloning toolkits (13 - 19), which leverage aligned plasmid parts based on the ordered pairings of restriction site overhangs to enable innumerable mix-and-match plasmid assembly options. However, such modular genetic tools have not yet been applied to most marine bacterial species. Thus, adapting and applying standardized molecular cloning tools for studying marine bacteria can provide a framework for addressing functional questions for basic science and biotechnology.

Marine bacteria are of specific interest as targets for genetic tool development due to their ability to produce diverse bioactive metabolites (20), their prominent associations in aquatic microbiomes, and their involvement in host-microbe symbioses (21 - 23). Alphaproteobacteria and Gammaproteobacteria, in particular, are the most abundant orders in the ocean (12) and are prominent members of the microbiomes of animals such as phytoplankton (12), tubeworms (21), and corals (24).

Of particular interest as targets for genetic manipulation are marine *Pseudoalteromonas* species because they produce a number of bioactive secondary metabolites (8, 25 - 29) and are often found in association with marine invertebrates (30 - 36). *Pseudoalteromonas* species are known to engage in a transient symbiosis called bacteria-stimulated metamorphosis, whereby surface-bound bacteria promote the larval-to-juvenile life cycle transition in invertebrates such as tubeworms and corals (37, 38). *Pseudoalteromonas luteoviolacea* stimulates the metamorphosis of the tubeworm *Hydroides elegans* (39, 40) by producing syringe-like protein complexes called Metamorphosis-Associated Contractile structures (MACs). MACs stimulate tubeworm metamorphosis by injecting an effector protein termed Mif1 into tubeworm larvae (40 - 42). Genes encoding the MACs structure are found in the *P. luteoviolacea* genome as a gene cluster encoding structural components, such as the *macB* baseplate and *macS* sheath, as well as the protein effector gene *mif1* (41). Despite the significant insights gained by using genetics in *P. luteoviolacea*, new genetic tools are needed to further dissect the function of MACs and their stimulation of tubeworm metamorphosis.

In this work, we utilize a modular plasmid toolkit, and contribute new Marine Modification Kit (MMK) plasmid parts, to study bacteria-stimulated metamorphosis in the Gammaproteobacterium, *P. luteoviolacea*. We demonstrate the broader utility of this approach by conjugating MMK plasmids into marine Alphaproteobacteria and Gammaproteobacteria that have been shown previously to be involved in diverse host-microbe interactions.

## RESULTS

### Toolkit-enabled quantitative promoter expression in *P. luteoviolacea*

**Fig 1:**
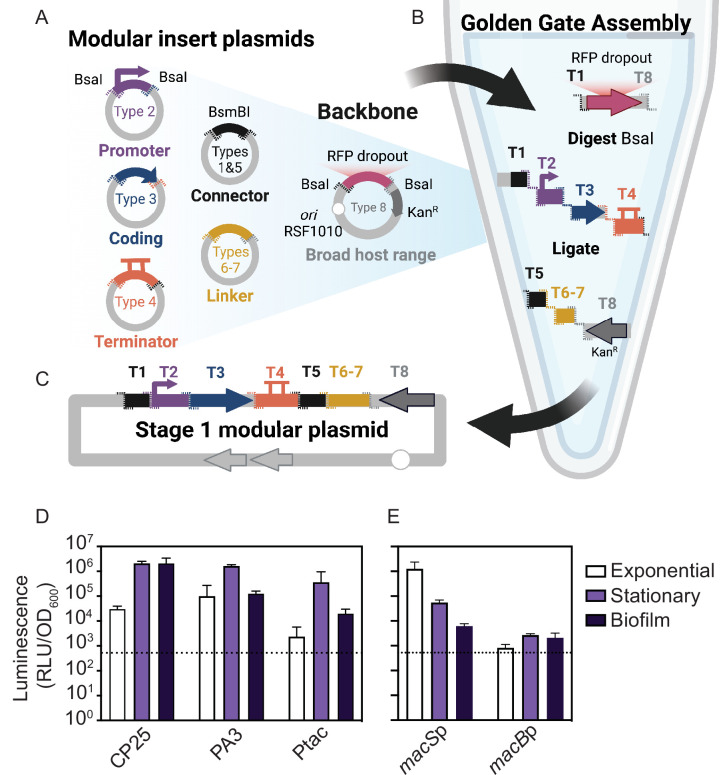
Schematic overview of the modular plasmid system and quantitative promoter measurements. (**A**) Schematic representation of the modular golden gate assembly plasmid parts with flanking BsaI cut sites (dashed lines). Overlapping 4 bp overhangs are color coordinated. The modular broad-host-range (BHR) backbone (pBTK402) contains inverted BsaI cut sites and an RFP dropout. (**B**) Golden Gate Assembly is performed in a one-tube reaction by digesting the backbone and insert part plasmids with BsaI and ligating with T4 ligase. (**C**) A modular stage-1 plasmid is complete when all overlapping inserts are successfully assembled in order. (**D and E**) Luciferase assays of *P. luteoviolacea* strains expressing plasmids with different promoters during exponential, stationary, or biofilm growth driving a Nanoluciferase (*NLuc*) gene where (**D**) shows CP25-*NLuc*-T7, PA3-*NLuc*-T7, Ptac-*NLuc*-T7 and (**E**) compares native MACs *macS* and *macB* promoters. Luminescence, as relative luminescence units (RLUs), is normalized to optical density at 600 nm (OD_600_) and plotted on a log base 10 scale. The dashed line indicates *P. luteoviolacea* cells expressing a non-luminescent plasmid as represented by the dotted line (*Y* = 524 RLU/OD_600_). Plotted is the mean of three biological replicates. Error bars indicate standard deviations.

To test the application of modular genetic tools in marine bacteria, we identified a set of preexisting parts from the Yeast Toolkit and Bee Toolkit platforms (17, 18) and used Golden Gate Assembly (14) for rapid, modular construction of plasmids (Fig. 1A through C). Each type of part is defined by its functional role (e.g., promoter and coding sequence [CDS]) and directional 4 bp overhangs generated by flanking Type IIS (BsaI) restriction sites. The modular parts include Type-1 and Type-5 stage-2 connectors with BsmBI recognition sites (17, 18), a Type-2 promoter with ribosome binding site (RBS), a Type-3 protein CDS (e.g., *gfp* and *Nanoluciferase*), a Type-4 terminator, an optional Type-6 repressor and Type-7 promoter with RBS, and a Type-8 backbone. Preexisting Type-8 backbones are available with different origins of replication (ColE1 and RSF1010) and antibiotic resistance markers (ampicillin, kanamycin, or spectinomycin resistance) (17, 18). For this work, we selected a broad-host-range (BHR) plasmid backbone containing a kanamycin resistance gene, a reporter CDS (fluorescent *gfp-*optim1, *mRuby*, or *Nanoluciferase* [*NLuc*]), T7 terminator, and a stage-2 assembly connector. The backbone selected has an RSF1010 origin of replication which is known to replicate in a broad range of Gram-positive and Gram-negative bacterial hosts at a copy number of 10–12 per chromosome and also contains a promiscuous origin of transfer and the plasmid-encoded mobilization genes *repA*, *repB*, *repC*, and *mobC* (43, 44). An auxotrophic MFDλ*pir* strain was used as the *E. coli* donor, thus obviating the need to generate antibiotic-resistant recipient strains to counter select *E. coli* donor cells after conjugation (45).

To apply the modular genetic tools in a marine symbiosis model, we tested the expression of five promoters in *P. luteoviolacea*. We assembled plasmids with each promoter fused to *NLuc* and conjugated the plasmids into *P. luteoviolacea*. We utilized two existing BHR promoters, PA3 and CP25, previously shown to work in diverse bee gut microbes (17, 46, 47). We also created a Ptac *lacO* promoter part (pMMK201), which is a hybrid of the *lac* and *trp* promoters amplified from the pANT4 plasmid (48). When *P. luteoviolacea* with the plasmids were grown in exponential, stationary, or biofilm growth phases, we observed at least 10-fold more luminescence signal compared to the background with all BHR promoters tested (Fig. 1D).

Previous observations have shown that the production of MACs is greatest during the exponential phase of growth when *P. luteoviolacea* is cultured in rich media (40). However, the expression of *mac* genes in live cultures has not been previously quantified. To observe the expression of two native *mac* promoters, we constructed two plasmids with *P. luteoviolacea* promoters driving the expression of the MACs structural genes; promoters from the MACs sheath (*macS* promoter*,* pMMK203) and baseplate (*macB* promoter, pMMK202) genes. The *macS*p luciferase reporter strain was elevated 1,000-fold in exponential growth as compared to 100-fold in stationary and 10-fold in biofilm phase, when compared to the detection limit (Fig. 1E). In contrast, the *macB*, baseplate promoter exhibited similar levels of luminescence among each phase, approximately 10-fold higher than the detection limit (Fig. 1E).

### Functional CRISPRi knockdown of secondary metabolite biosynthesis in *P. luteoviolacea*

**Fig 2:**
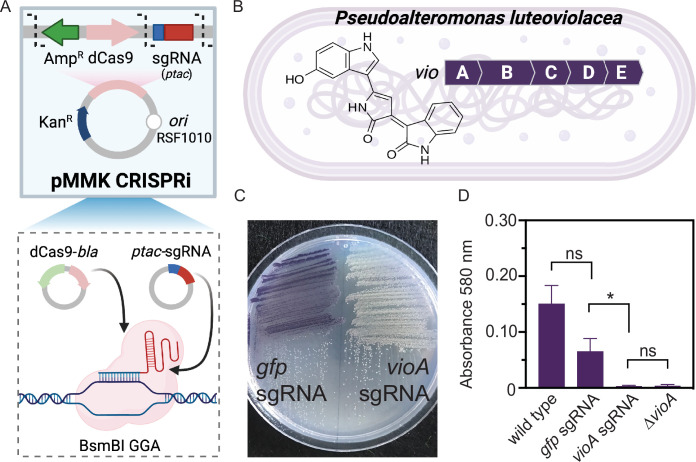
CRISPRi knockdown of secondary metabolite production in *P. luteoviolacea.* (**A**) Schematic representation of modular CRISPRi parts adapted to include dCas9-*bla* and Ptac sgRNA parts, pMMK601, and pMMK602, respectively. Part plasmids are combined, and a BsmBI Golden Gate Assembly was performed. (**B**) Schematic representation of the violacein gene cluster *vioABCD* in *P. luteoviolacea* and the violacein molecular structure. The CRISPRi system was assembled with an sgRNA targeting the *vioA* gene (pMMK603) and employed to knock down violacein production in *P. luteoviolacea*. (**C**) *P. luteoviolacea* with *gfp* (pMMK815) or *vioA* (pMMK816) sgRNA plasmids grown on marine agar plates. (**D**) Quantification of violacein production (measured at 580 nm) between *P. luteoviolacea* containing *gfp* or *vioA* sgRNA plasmids. Asterisks indicate significant differences (*P = 0.02, Dunn’s multiple comparisons test). Bars represent the mean of eight total replicates and error bars indicate standard deviations.

While previous studies in *P. luteoviolacea* have used gene knockouts to interrogate gene function, these approaches are time-consuming and low-throughput. We therefore tested whether *P. luteoviolacea* is amenable to gene knockdown via CRISPR interference (CRISPRi) (Fig. 2A and B) (49, 50). As a proof of concept, we targeted the *vioA* gene that encodes a key enzyme in the biosynthesis of violacein (51), which gives *P. luteoviolacea* its characteristic purple pigment (Fig. 2B). An assembled plasmid containing dCas9 and a single-guide RNA (sgRNA) targeting *vioA* (pMMK603) was conjugated into *P. luteoviolacea* resulting in the visible absence of the purple pigment associated with violacein production on the plate (Fig. 2C). We also created a plasmid containing dCas9 and a sgRNA targeting *gfp* to test whether the presence of the CRISPRi machinery adversely affected wild-type (WT) *P. luteoviolacea* or violacein production. No difference was observed between the growth and cell morphology of *P. luteoviolacea* containing *gfp* or *vioA* sgRNA CRISPRi plasmids compared to WT (Fig. S1). WT *P. luteoviolacea* produced violacein as expected, while *P. luteoviolacea* with CRISPRi with the *gfp* sgRNA produced a statistically comparable amount of violacein (adjusted *P* = 0.26, *n* = 8, Dunn’s multiple comparison test). A significant reduction of violacein production was observed between cultures of *P. luteoviolacea* strains expressing the *vioA* and *gfp* targeting CRISPRi plasmids (adjusted *P* = 0.02, *n* = 8, Dunn’s multiple comparison test) (Fig. 2D). The lack of violacein in the *vioA* knockdown strain was comparable to that of a *P. luteoviolacea* strain with an in-frame deletion of *vioA* (adjusted *P* = 0.26, *n* = 8, Dunn’s multiple comparison test) (Fig. 2D). These results demonstrate the successful implementation of CRISPRi for gene knockdown in *P. luteoviolacea*.

### Functional CRISPRi knockdown and visualization of *P. luteoviolacea* during a tubeworm-microbe interaction

**Fig 3:**
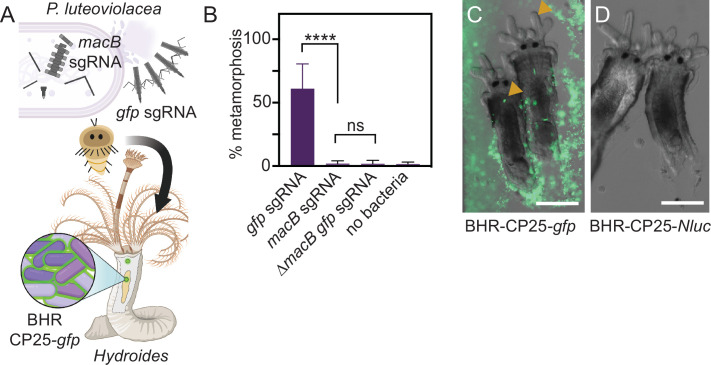
Functional knockdown of MACs and visualization of *P. luteoviolacea* during the tubeworm-microbe interaction. (**A**) Schematic depicting *P. luteoviolacea* and the production of MACs, which induce tubeworm metamorphosis. CRISPRi single-guide RNA (sgRNA) targeting the *macB* MACs baseplate gene prevents MACs from assembling, rendering the bacterium unable to induce metamorphosis. Cells that produce intact MACs are able to induce tubeworm metamorphosis. A strong fluorescent reporter strain (BHR-CP25-*gfp*) enabled visualization of live tubeworm-bacteria interactions. (**B**) Bar graph representing biofilm metamorphosis assays with *P. luteoviolacea* carrying a CRISPRi plasmid targeting *macB* or *gfp* and *Hydroides* tubeworms. A *P. luteoviolacea* ∆*macB* strain with a sgRNA targeting *gfp* and a treatment without bacteria (no bacteria) were included as controls. Biofilm concentrations were made with cells at OD_600_ 0.1. Bars plotted show the average of 12 replicates, performed across three independent experiments. Each well contained 20–40 worms. Error bars indicate standard deviations. Statistical significance between treatments was determined by Dunn’s multiple comparisons test (*N* = 12). (**C and D**) Merged fluorescence and DIC micrographs of *Hydroides elegans* juveniles imaged 24 h after the competent larvae were exposed to inductive biofilms of *P. luteoviolacea* containing plasmids with (**C**) CP25-*gfp* or (**D**) CP25-*NLuc*. Strains containing *NLuc* plasmids were used as a negative control to account for autofluorescence. Yellow arrows show accumulation of fluorescent bacteria in the *Hydroides* juvenile pharynx. Scale bar is 100 µm.

We next tested whether CRISPRi would be functional in the context of a marine host-microbe interaction by targeting the *macB* gene, which encodes the MACs baseplate, an essential component of the MACs complex that induces tubeworm metamorphosis (39, 40) (Fig. 3A). Biofilm metamorphosis assays were performed comparing *P. luteoviolacea* strains with sgRNAs targeting *macB* (pMMK604) or the sgRNA targeting *gfp* control (Fig. 3B). The strain with sgRNA targeting *macB* exhibited significantly reduced levels of tubeworm metamorphosis compared to the *gfp-*sgRNA control (adjusted *P* < 0.0001, Dunn’s multiple comparisons test, *n* = 12) (Fig. 3B). The reduction of metamorphosis stimulation in the *macB*-sgRNA knockdown strain was comparable to that of a *P. luteoviolacea* strain with an in-frame deletion of *macB* carrying the *gfp-*sgRNA control plasmid (adjusted *P* ≥ 0.99, Dunn’s multiple comparison test, *n* = 12) (Fig. 3B). These results demonstrate that CRISPRi paired with a modular plasmid system is a viable tool for interrogating gene function during a marine host-microbe interaction.

To date, bacteria have not been visualized during or after the stimulation of metamorphosis in *Hydroides*. To test whether marine bacteria harboring a toolkit plasmid are amenable to live-cell imaging when in association with juvenile tubeworms, we created biofilms of *P. luteoviolacea* containing plasmids encoding CP25-*gfp-*T7 (*gfp*) or CP25-*Nanoluc-*T7 (*NLuc*) and added competent *Hydroides* larvae. After incubation for 24 h, biofilms of *gfp*-expressing *P. luteoviolacea* were clearly observed when visualized by fluorescence microscopy (Fig. 3C). *P. luteoviolacea* stimulated *Hydroides* metamorphosis while carrying a modular plasmid and fluorescent bacteria were observed being ingested by the *Hydroides* juveniles. Bacteria can be seen collecting in the pharynx (Fig. 3C, yellow arrows), then moving in a peristaltic fashion toward the gut (Movie S1). In contrast, bacteria containing a CP25-*NLuc-*T7 plasmid were difficult to visualize by light microscopy, in the absence of the *gfp* fluorescent marker (Fig. 3D). Taken together, the modular plasmid system enables live imaging and experimentation during a marine host-microbe interaction.

### Applying the modular toolkit in marine Alphaproteobacteria and Gammaproteobacteria

**Fig 4:**
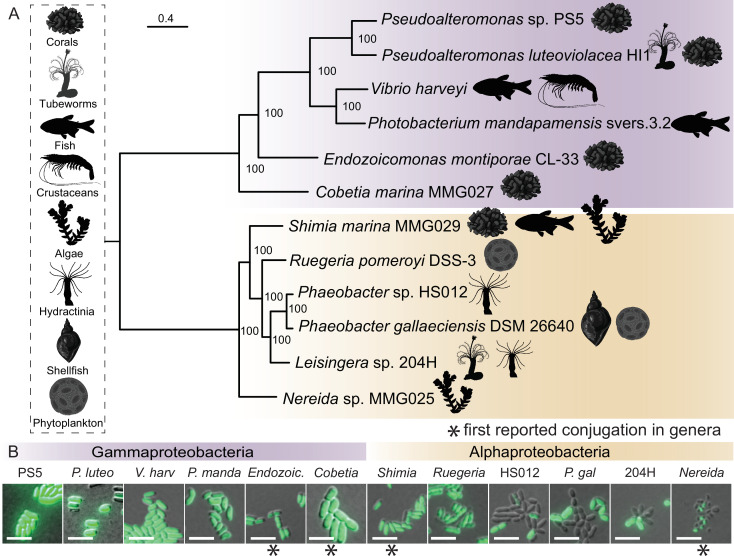
Marine Proteobacteria are amenable to plasmid uptake and stable replication of toolkit plasmids. (**A**) Maximum likelihood phylogeny built using the whole genomes of 12 strains selected for manipulation and successfully conjugated in this study (52, 53). All strains used in this study are known for their interaction with a range of marine biota and the icons depicting their associated host are shown in the vertical box. Gammaproteobacteria strains are highlighted in purple and Alphaproteobacteria strains are shown in gold. Scale bar is 0.4 and bootstraps were generated using the rapid-bootstrapping method (54). The tree was rooted at the midpoint with FigTree (v1.4.4). (**B**) Fluorescence and DIC overlay micrographs of overnight cultures containing constitutively expressed RSF1010 *ori* fluorescence vector (CP25-*gfp*-T7). Scale bar is 5 µm. Stars denote environmental strains that serve as the first reported conjugation for that genera.

Given the success of genetic manipulation of *P. luteoviolacea*, we tested whether other marine Proteobacteria might be amenable to conjugation and retention of a modular genetic toolkit plasmid. To this end, we isolated or acquired representative bacteria that are known to engage in symbioses with marine plants or animals in the ocean (Fig. 4A; Tables S1 and S2). To enable genetic selection using antibiotics, we determined the minimum inhibitory concentration for each bacterial strain tested against kanamycin (Table S1). When conjugation was performed using the BHR (RSF1010) plasmid backbone, CP25 promoter, *gfp* reporter, and T7 terminator, we observed the expression of *gfp* in 12 marine strains across two proteobacterial classes, four orders, and 10 genera (Fig. 4B). Adaptations to the conjugation protocol and the use of constitutive promoters driving *gfp* enabled visual confirmation of successful conjugation (Fig. 4B, Materials and Methods).

## DISCUSSION

### Modular genetic tools provide insights about bacteria-stimulated metamorphosis

We tested a modular plasmid toolkit on a genetically tractable marine bacterium, *P. luteoviolacea*, that promotes the metamorphosis of the tubeworm *Hydroides elegans* (40, 41, 55) and produces several bioactive secondary metabolites (26, 29, 56, 57). We expand the tools available for functional interrogation of bacteria-stimulated metamorphosis in *P. luteoviolacea* by quantifying gene expression by a luminescence assay (Fig. 1D and E), and using CRISPRi to knock down the secondary metabolite, violacein (Fig. 2C and D), as well as a metamorphosis-associated gene, *macB* (Fig. 3B) during the bacteria-tubeworm interaction. Distinct patterns of sheath (*macS*p) (41, 58) and baseplate (*macB*p) promoter induction suggest distinct mechanisms of gene regulation within the MACs gene cluster. Expression of the sheath gene was sensitive to bacterial mode of growth, while baseplate gene expression appeared static across the growth conditions tested. Although MACs are known to produce two effectors that stimulate tubeworm metamorphosis and kill eukaryotic cells (41, 58), the environmental conditions that promote MACs production remain poorly characterized. The tools developed here could help to characterize the conditions under which *P. luteoviolacea* MACs are produced or assembled and could help in the development of MACs or other contractile injection systems for use in biotechnology (59, 60). The modular tools in this work open new capabilities for interrogating bacteriology, including the ability to quantify gene expression in live cultures, knock down gene expression for rapid functional testing, and visualize bacteria during an *in vivo* interaction.

Whether, and how, bacteria and the animal are harmed or benefit from the interaction during bacteria-stimulated metamorphosis remains a prominent question in the field (38, 61, 62). Swimming *Hydroides* larvae initially encounter and are stimulated to undergo metamorphosis by the bacterial biofilm. And MACs were previously visualized within *P. luteoviolacea* biofilms by tagging the MACs baseplate with super-folder GFP (40). However, less attention has been put on the interaction between *Hydroides* and the bacteria after metamorphosis. Previous work by Gosselin et al. has shown that *Hydroides* is able to feed on bacteria as the sole food source (63). In the present work, we visualize live bacteria surrounding and being ingested by *Hydroides* juveniles (Fig. 3C) (21). The visualization of transgenic bacteria in *Hydroides* will enable future lines of research that can help dissect the role of microbiome seeding in bacteria-stimulated metamorphosis. More broadly, our results showcase the feasibility of using a modular plasmid toolkit to test hypotheses about bacteria-stimulated metamorphosis and provide a framework for the interrogation of other bacteria and their products that promote host-microbe symbioses (36, 64, 65).

### Toolkit compatibility in marine bacteria

In this work, we explore genetic tractability in 12 ecologically relevant marine bacteria that belong to two Proteobacterial classes (Fig. 4). The Gammaproteobacteria strains conjugated successfully in this study are a selection of symbiosis-associated strains representing five genera (Fig. 4A) (66 - 72). To our knowledge, this is the first report of genetic tractability in strains from the genera *Endozoicomonas*, *Nereida*, and *Cobetia* (Fig. 4B). *Endozoicomonas* species are among the most abundant bacterial symbionts in some corals and other marine hosts (73 - 75). Related strains of *Cobetia* have been implicated in thermotolerance against bleaching in coral experiments with probiotic consortium treatments (76). The conjugation of the representative *Endozoicomonas* and *Cobetia* strains in this study is a considerable step toward exploring function in coral host-microbiome interactions at a critical time to encourage the restoration of coral reefs (6, 77, 78). The genetic conjugation of *Pseudoalteromonas* sp. PS5 in this study presents an opportunity to explore secondary metabolite production, including the coral metamorphosis-inducing compound, tetrabromopyrrole (Fig. 4) (36, 79). The Alphaproteobacteria strains tested for compatibility with MMK plasmids fall within the *Roseobacter* group (Fig. 4A), an ecologically important group of bacteria known to play a role in sulfur and carbon cycling on marine phytoplankton (80 - 82). *Roseobacter* strains have also been explored as probiotics for the aquaculture industry (83 - 85). We tested the toolkit with the tractable, phytoplankton-associated species of *Phaeobacter gallaeciensis* (86), and *Ruegeria pomeroyi* (87), and demonstrated conjugation with invertebrate microbiome-associated strains *Phaeobacter* sp. HS012 (88) and *Leisingera* sp. 204H (89) (Fig. 4). Using modified *Shimia* sp. may be of interest for future coral microbiome studies (90 - 93). Species in the *Nereida* genus have been isolated from kelp (94) and are associated with gall formations (95, 96). Tractability in this strain could help guide further understanding of microbe-seaweed interactions (97, 98), kelp aquaculture, and the development of kelp probiotics (99). In summary, the development of methods and established tractability of several new strains and genera have significant implications for the future of bacterial genetic development in established and emerging symbiosis systems.

### Future modifications

The modularity of the plasmid toolkit enables the potential for creating new plasmids that are compatible with the existing system to boost functionality. For example, the addition of backbone plasmid parts (Type-8) with different origins of replication and selectable markers could allow utilization in bacteria that are naturally resistant to the antibiotics used in this and prior works (17, 18). We have created a Type-8 Tn10 transposon backbone for stable integration of toolkit parts into the genomes of marine bacteria and used this part to integrate a fluorescent *gfp* marker into the genome of *Pseudoalteromonas* sp. PS5 (79). Type-8 parts like this could be used to tag and track marine bacteria for studying host-microbe interactions in the future.

The current promoter driving dCas9 is constitutive. However, adding an inducible promoter driving dCas9 (e.g., P_BAD_ for arabinose induction) would allow the CRISPRi system to be controllable. The expression of *gfp* was not uniformly observed in the *Phaeobacter*, *Leisingera*, and *Nereida* strains (Fig. 4B). However, the plasmid toolkit could be used to identify plasmid components that would produce uniform expression (e.g., different origins of replication, selectable markers, promoters, etc.). In the future, more strains may be tested for manipulation with the present toolkit plasmids for applying genetics in a broader array of bacteria types.

### Conclusion

The modular plasmid toolkit described here provides a basis for streamlining the genetic manipulation of marine bacteria for basic and applied purposes. These tools reveal new possibilities to study marine microbes in the context of plant and animal interactions, or with challenging and diverse non-model bacteria, ultimately helping us harness marine microbes for research, bioproduction, and biotechnology.

## MATERIALS AND METHODS

### Bacterial culture

A list of strains used in this study, isolation sources, accession numbers, and minimum inhibitory concentration can be found in Table S1. Environmental strains of marine bacteria were isolated and cultured on Marine Broth (MB) 2216 (BD Difco) and or natural seawater tryptone (NSWT) media (1 L 0.2 µm filtered natural seawater from Scripps Pier, La Jolla, CA, 2.5 g tryptone, 1.5 g yeast extract, 1.5 mL glycerol). MB and NSWT media are used interchangeably throughout the study; however, the experiments were always conducted using only one media type. Marine bacteria were incubated between 25°C and 30°C, and cultures were shaken at 200 rpm. All liquid cultures were inoculated with a single colony and incubated between 16 and 18 h, unless otherwise indicated. *E. coli* SM10λ*pir* and S17-1λ*pir* were cultured in LB (Miller, BD Difco) at 37°C, shaking at 200 rpm. *E. coli* MFDλ*pir* (45) was cultured in LB supplemented with 0.3 mM Diaminopimelic acid (DAP). For *E. coli,* antibiotic selections with ampicillin, kanamycin, and chloramphenicol were performed using a concentration of 12.5 µg mL^−1^.

### Plasmid construction and assembly

Golden Gate Assembly-compatible parts from the BTK, YTK (17, 18), and MMK used in this work can be found in Table S3. New plasmid parts were made by PCR amplifying insert and backbone fragments and combining them with Gibson Assembly with a 2:1 ratio (20 fmol insert: 10 fmol backbone) (100). PCR amplification was performed with custom primers (Table S4), a high-fidelity DNA polymerase (PrimeSTAR GXL, Takara), and purified using a DNA Clean and Concentrator kit (Zymo Research). Part plasmids were assembled to make a stage 1 plasmid using Golden Gate Assembly, with T4 DNA ligase (Promega) and either BsaI or BsmBI (New England Biolabs), depending on the construct. Single-tube assembly was performed by running the following thermocycler program (BsaI/BsmBI): 37/42°C for 5 min, 16°C for 5 min, repeat 30×, 37/55°C for 10 min, and 80°C for 10 min. The assemblies were directly electroporated into S17-1λ*pir* cells, confirmed by colony PCR (EconoTaq PLUS Green, LGC Biosearch) with internal primers, and then electroporated into MFDλ*pir* cells for conjugation. To facilitate assembly for and expression of CRISPRi parts in *P. luteoviolacea*, we moved the BsmBI cut site in the dCas9 part plasmid (pBTK614) to a location where the existing *bla* gene will be retained in the assembled plasmid (pMMK601), and thus also conferring resistance to ampicillin. In the sgRNA plasmid (pBTK615), we replaced the existing PA1 promoter with the Ptac promoter (including −35 and −10 sequences but excluding *lacO*), which drives the sgRNA expression (pMMK602). The CRISPRi assemblies were electroporated directly into SM10λ*pir* cells and shuttled to MFDλ*pir* cells for conjugation.

### Biparental conjugation in marine bacteria

*E. coli* donor strains (MFDλ*pir* or SM10λ*pir*) containing the mobilizable plasmids were grown under antibiotic selection in LB with the appropriate supplements (including 0.3 mM DAP for *E. coli* MFDλ*pir*). Conjugations were performed as previously described (17) with modifications for culturing marine bacteria. Briefly, several colonies of the recipient strains were inoculated and grown overnight in liquid culture. Recipient and donor cultures were spun down (4,000 × *g* for 2 min) in a 1:1 OD_600_ ratio. All donor supernatant was removed leaving only the cell pellet. All but 100 µL of the recipient supernatant is removed, and the cell pellet is resuspended. The recipient suspension was transferred to the donor pellet, which was resuspended with the recipient cells. Two 50 µL spots are plated onto NSWT (supplemented with 0.3 mM DAP for MFDλpir-mediated conjugations) and incubated overnight at 25°C with the lids facing up. The next day, spots were scraped up with a pipette tip and resuspended in 500 µL of liquid marine media and 100 µL was plated onto marine media containing antibiotic selection, according to the minimum inhibitory concentration (Table S1). Streptomycin-resistant *P. luteoviolacea* (Table S1) were conjugated with *E. coli* SM10λ*pir*, and counterselection was performed with 100–200 µg/mL streptomycin. All other marine bacteria (Table S1) were conjugated with *E. coli* MFDλ*pir*, and transconjugant selection was performed in the absence of DAP. Several of the bacteria take longer to grow or do not reach a high optical density (i.e., *Endozoicomonas*, *Ruegeria*, and *Nereida*) in culture. Slower-growing marine bacteria were conjugated by growing larger 50 mL initial volumes of culture and spinning down the entire culture to reach 1:1 (donor:host) ratios.

### Phylogeny

Strains or close representative strains used in this study were compiled into a genome group on PATRIC v3.6.12 (101). A whole genome phylogenetic codon tree composed of 100 single-copy genes (102) was performed using the Phylogenetic Tree Service (103 - 105). A maximum likelihood phylogeny was generated using the best protein model found by RaxMLv8.2.11 (106), which was LG. Bootstraps were generated using the rapid bootstrapping algorithm with the default of 100 resamples (54). The tree was visualized with FigTree v1.4.4. and was rooted at the mid-line.

### Growth curve

*Pseudoalteromonas luteoviolacea* ∆*vioA* and WT were grown on MB agar plates and incubated overnight at 25°C. *P. luteoviolacea* strains expressing CRISPRi plasmids were grown on MB agar plates with 200 µg mL^−1^ of kanamycin and grown overnight at 25˚C. Single colonies were picked and inoculated into 5 mL of MB liquid media with the respective antibiotics listed above. Two biological replicate cultures were inoculated for each strain by picking different colonies from the agar plate and inoculating separate 5 mL cultures. Cultures were incubated at 25°C for 18 h shaking at 200 rpm. From the initial cultures, a subculture was created by performing a 1:25 dilution into the subculture. The subculture consisted of 25 mL of MB liquid media and 1 mL of original culture along with the respective antibiotics into a 125-mL flask. Subcultures were incubated at 25°C shaking at 200 rpm throughout the growth curve experiment. Optical density (OD) at a wavelength of 600 nm was measured from the subculture every half hour for the first 5 h and then measured every hour until 10 h with a final measurement at 24 h.

### Luciferase culture and assay

*P. luteoviolacea* containing plasmids with constitutive or native promoters driving *Nanoluciferase* (*NLuc*) were inoculated into 5 mL of MB or NSWT media with appropriate antibiotics and grown at 25°C at 200 rpm for 24 h. Each biological replicate was represented by a separate culture. Cultures used for the growth phase assay were inoculated as a 1:100 dilution with the appropriate antibiotic, and then incubated at 25°C and shaking at 200 rpm. The luminescence of cultures was measured at exponential (OD_600_ 0.35–1.0), early stationary (OD_600_ 1.0–1.45), or late stationary (OD_600_ 2.38–2.54) phases. For biofilm cultures, 1.5 mL of stationary-phase culture was pelleted and plated as a single spot on NSWT or MB plates. Biofilm plates were incubated at 20–25°C for 24–28 h. Each spot was scraped with a pipette tip and resuspended in 200 µL of NSWT or MB media before being resuspended in NSWT or MB. Luciferase reactions were performed with 100 µL of bacterial culture or biofilm resuspension aliquoted into opaque white walled 96-well plates (Corning #3642), with a modified protocol as written for Promega Nano-Glo Live Cell Assay System (Promega, catalog #N2011). Briefly, bacteria and the final reagent mix (2.5 µL of Nano-Glo LCS dilution buffer, 0.5 µL of Nano-Glo live cell substrate, and 17.5 µL of water) were read at a 1:1 ratio. Luminescence was measured on a Molecular Devices Microplate FilterMax F5 reader with a custom program on the Softmax Pro 7 software. Readings were done on the kinetic luminescence mode at 2 min intervals for 20 min in total, using a 400-ms integration time, a 1-mm height read, and no other optimization or shaking settings. The detection limit is defined as the average expression of *P. luteoviolacea* cells expressing a non-luminescent plasmid across growth conditions. Raw data were normalized to the OD_600_ of the culture used and plotted with an *N* = 3 biological replicates.

### Violacein extraction

The specified *P. luteoviolacea* strains were struck onto NSWT media containing 200 µg mL^−1^ of streptomycin and kanamycin and incubated overnight at 25°C. Single colonies were inoculated into 5 mL of liquid media containing the same antibiotic concentrations. Cultures were incubated at 25°C, shaking at 200 rpm between 18 and 20 h. Cultures were removed from the incubator and standardized to an OD_600_ of 1.5. The cells were pelleted, and the supernatant was removed. The cell pellet was resuspended in 200 µL of 100% ethanol. The resuspended cells were pelleted and the supernatant containing the crude extract was recorded on a BioTek Synergy HT plate reader (Vermont, USA) using the Gen5 program (v2.00.18) with an endpoint reading at 580 nm.

### Microscopy

Microscopy was performed using a Zeiss Axio Observer.Z1 inverted microscope equipped with an Axiocam 506 mono camera and Neofluar10x/0.3 Ph1/DICI (*Hydroides* co-cultures) or Apochromat 100×/1.4 Oil DICIII (bacteria only) objectives. The Zeiss HE eGFP filter set 38 was used to capture GFPoptim-1 expression and Zeiss HE mRFP filter set 63 was used to capture *mRuby2* expression. For *Nanoluciferase* controls, images were captured using the same fluorescence exposure times as the *gfp* optim-1 and *mRuby2* labeled strains of the same species.

Bacterial culture (2 µL) was added to freshly prepared 1% saltwater low-melt agarose (Apex catalog #20-103, Bioresearch products) pads on glass slides and coverslips were placed on top. *Hydroides elegans* were prepared in visualization chambers (Lab-Tek Chambered Coverglasses catalog #155411PK) with bacteria and imaged.

### *Hydroides elegans* culture

*Hydroides elegans* adults were collected from Quivira Basin, San Diego, CA, USA. The larvae were cultured and reared as previously described (40, 107). Larvae were maintained in beakers containing filtered artificial seawater (35 PSU) and were given new beakers with water changes daily. The larvae were fed live *Isochrysis galbana* and cultures were maintained as described previously. The larvae were used for metamorphosis assays once they reached competency (between 5 and 7 d old) (108).

### *Hydroides elegans* metamorphosis assays

Biofilm metamorphosis assays were performed using previously described methods (39, 40, 109). Briefly, bacteria were struck onto MB plates with 300 µg mL^−1^ kanamycin as appropriate and were incubated overnight at 25°C. Up to three single colonies were inoculated into liquid broth and incubated overnight (between 15 and 18 h), shaking at 200 rpm. Cultures were pelleted at 4,000 × *g* for 2 min, the spent media were removed, and the cell pellets were washed twice with filtered artificial sea water (ASW). The concentration of the cells was diluted to OD_600_ of 0.1, and four 100 µL aliquots of the cell concentrate were added to 96-well plates. The cells were given between 2 and 3 h to form biofilms, then the planktonic cells were removed and the adhered cells were washed twice with filtered ASW. Between 20 and 40 larvae were added to each well in 100 µL of filtered ASW. Metamorphosis was scored after 24 h. Three biological replicates were performed on different days using separate *Hydroides* larvae originating from different male and female animals.

Chambered metamorphosis assays were performed using the same preparation principles as described above with the following modifications. Visualization chambers (Lab-Tek, catalog # 155411) were used for setting up the metamorphosis assay, then subsequently imaged. Inductive strains containing constitutively expressed *gfp*/*mRuby*/*NLuc* plasmids were struck out onto MB media containing 300 µg mL^−1^ kanamycin. Several colonies were inoculated into 5 mL MB media with antibiotics. Cultures were grown for 18 h and cells were washed and allowed to form biofilms as described above. Cell concentrations ranging between OD_600_ 0.1 and 0.5 were used to elicit optimal metamorphosis. Larvae were concentrated and the resident filtered ASW was treated with 300 µg mL^−1^ kanamycin. Larvae were imaged 24 h later.

### Online protocols

Selected protocols used in this study can be accessed on the Shikuma Lab protocols.io page: https://www.protocols.io/workspaces/shikuma-lab-sdsu (110 - 112). 

## SUPPLEMENTAL MATERIAL

Supplemental material mbio.01502-23-s0001.docx Tables S1 to S4, Fig. S1, and Movie S1 legend.

Movie S1 mbio.01502-23-s0002.mov Hydroides feeding.

ASM does not own the copyrights to Supplemental Material that may be linked to, or accessed through, an article. The authors have granted ASM a non-exclusive, world-wide license to publish the Supplemental Material files. Please contact the corresponding author directly for reuse.
