# Supplementary material for: A modular plasmid toolkit applied in marine bacteria reveals functional insights during bacteria-stimulated metamorphosis
Source: mBio. 2023 Aug 2;14(4):e01502-23. doi: 10.1128/mbio.01502-23 (PMC10470607; doi:10.1128/mbio.01502-23)
Supplement: Supplemental material — Tables S1 to S4, Fig. S1, and Movie S1 legend. [file mbio.01502-23-s0001.docx]

**SUPPLEMENTARY MATERIAL**

**A modular plasmid toolkit applied in marine bacteria reveals functional insights during bacteria-stimulated metamorphosis**

**AUTHORS**

Amanda T. Alker^1^, Morgan V. Farrell^1^, Alpher E. Aspiras^1^, Tiffany L. Dunbar^1^, Andriy Fedoriouk^1^, Jeffrey E. Jones^1^, Sama R. Mikhail^1^, Gabriella Y. Salcedo^1^, Bradley S. Moore^2^ and Nicholas J. Shikuma^1^#

#Corresponding author: Nicholas J. Shikuma (nshikuma@sdsu.edu)

**INSTITUTIONS**

**1** Department of Biology,

San Diego State University, San Diego, CA 92182 USA

**2** Center for Marine Biotechnology and Biomedicine, Scripps Institution of Oceanography, University of California, San Diego, La Jolla, CA 92093 USA

**Table S1. List of strains used in this study.** MIC = minimum inhibitory concentration. Kan = kanamycin. Str = streptomycin. NT = antibiotic sensitivity not tested.

| **Strain no.** | **Strain** | **Genotype** | **Class** | **Order** | **MIC Kan (µg/mL)** | **Source** |
| --- | --- | --- | --- | --- | --- | --- |
| NJS005 | *Pseudoalteromonas luteoviolacea* HI1 | StrR | Gamma | *Alteromonadales* | 200 | (39) |
| NJS023 | *Pseudoalteromonas luteoviolacea* HI1 | StrR, ∆*macB* | Gamma | *Alteromonadales* | NT | (40) |
| NJS017 | *Pseudoalteromonas luteoviolacea* HI1 | StrR, ∆*vioA* | Gamma | *Alteromonadales* | NT | This study |
| NJS595 | *Pseudoalteromonas* sp. PS5 | Wild type | Gamma | *Alteromonadales* | NT | (36) |
| NJS597 | *Pseudoalteromonas* sp. PS5 | StrR | Gamma | *Alteromonadales* | 200 | This study |
| NJS445 | *Vibrio harveyi* | Wild type | Gamma | *Vibrionales* | NT | Stanley Maloy |
| MT002 | *Photobacterium mandapamensis* svers.3.2 | Wild type | Gamma | *Vibrionales* | 400 | Alison Gould |
| NJS662 | *Endozoicomonas montiporae* CL-33 | Wild type | Gamma | *Oceanospirillales* | 100 | (110) |
| NJS775 | *Cobetia* sp. MMG027 | Wild type | Alpha | *Oceanospirillales* | 200 | This study |
| NJS302 | *Shimia* sp. MMG029 | Wild type | Alpha | *Rhodobacterales* | 300 | This study |
| NJS409 | *Ruegeria pomeroyi* DSS-3 | Wild type | Alpha | *Rhodobacterales* | 300 | (87) |
| NJS491 | *Phaeobacter* sp. HS012 | Wild type | Alpha | *Rhodobacterales* | 300 | (88) |
| NJS408 | *Phaeobacter gallaeciensis* ATCC 700781 (DSM 26640) | Wild type | Alpha | *Rhodobacterales* | 300 | (86) |
| NJS204 | *Leisingera* sp. 204H | Wild type | Alpha | *Rhodobacterales* | 200 | (89) |
| NJS678 | *Nereida* sp. MMG025 | Wild type | Alpha | *Rhodobacterales* | 200 | (94) |
| **Strain no.** | **Strain** | **Genotype** |  |  |  | **Source** |
| pNJS488 | *Escherichia coli* S17-1pir | TpR SmR recA thi pro (rK− mK+) RP4: 2-Tc:Mu: Km Tn7 λpir | | | | (111) |
| NJS604 | *Escherichia coli* MFDλpir | MG1655 RP4-2-Tc::[ΔMu1::aac(3)IV-ΔaphA-Δnic35-ΔMu2::zeo] ΔdapA::(erm-pir) ΔrecA | | | | (45) |
| pNJS033 | *Escherichia coli* SM10λpir | thi thr leu tonA lacY supE recA::RP4-2-Tc::Mu Km λpir | | | | (112) |

**Table S2. List of strains tested for plasmid compatibility.** Strains were tested for compatibility by conjugation with *E. coli* MFDλpir with pBTK402-CP25-gfp-T7 plasmid. Strains were resistant to kanamycin, did not grow, or expression of *gfp* was not observed under the conditions tested.

| **Strain No.** | **Strain** | **Class** | **Order** | **Source** |
| --- | --- | --- | --- | --- |
| NJS462 | *Erythrobacter* sp. | *Alpha* | *Sphingomonadales* | This study |
| NJS677 | *Pseudoalteromonas piratica* sp*.* | *Gamma* | *Alteromonadales* | This study |
| NJS023 | *Salinimicrobium* sp. | *Gamma* | *Alteromonadales* | This study |
| NJS630 | *Shewanella* sp. MMG014 | *Gamma* | *Alteromonadales* | This study |
| NJS456 | *Winogradskyella* sp. | *Flavobacteriia* | *Flavobacteriales* | This study |
| NJS631 | *Aquimarina* sp. MMG015 | *Flavobacteriia* | *Flavobacteriales* | This study |

**Table S3. List of plasmids used in this study.** N/A = 5’ or 3’ restriction site not applicable. Amp = ampicillin. Kan = kanamycin. Str = streptomycin.

| **Plasmid** | **Type** | **5’ Site** | **3’ Site** | **Description** | **Marker** | **Origin** | **Source** |
| --- | --- | --- | --- | --- | --- | --- | --- |
| pBTK001 | Entry vector | N/A | N/A | Entry vector for generating new parts | CamR | p15A | (17) |
| pYTK008 | Connector | 1 | 1 | ConLS’ connector | CamR | ColE1 | (18) |
| pBTK107 | Promoter | 2 | 2 | CP25 promoter, RBS | CamR | ColE1 | (17) |
| pBTK121 | Promoter | 2 | 2 | PA3 promoter, RBS | CamR | p15A | (17) |
| pMMK201 | Promoter | 2 | 2 | Ptac *LacO^+^* promoter, RBS | CamR | ColE1 | This study |
| pMMK202 | Promoter | 2 | 2 | HI1 *macB* promoter, RBS | CamR | ColE1 | This study |
| pMMK203 | Promoter | 2 | 2 | HI1 *macS* promoter, RBS | CamR | ColE1 | This study |
| pYTK047 | GFP Dropout | 2 | 4 | *gfp* dropout (internal BsaI sites) | CamR | ColE1 | (18) |
| pBTK205 | Coding sequence | 3 | 3 | *gfp* optim-1 | CamR | ColE1 | (17) |
| pYTK034 | Coding sequence | 3 | 3 | *mRuby2* | CamR | ColE1 | (18) |
| pBTK206 | Coding sequence | 3 | 3 | *NanoLuc* | CamR | ColE1 | (17) |
| pBTK305 | Terminator | 4 | 4 | T7 terminator | CamR | ColE1 | (17) |
| pYTK073 | Connector | 5 | 5 | ConRE’ connector | CamR | ColE1 | (18) |
| pBTK402 | Origin, Marker | 8 | 8 | *rfp* dropout | KanR | RSF1010 | (17) |
| pBTK527 | Origin, Marker | ConLS' | ConRE' | BsmBI sites flanking spacer | KanR | RSF1010 | (17) |
| pBTK614 | dCas9 | ConL1 | ConRE | dead cas9 | AmpR | ColE1 | (17) |
| pMMK601 | dCas9-bla | ConL1 | ConRE | dead cas9 with *bla* (resistance gene) | AmpR | ColE1 | This study |
| pBTK615 | sgRNA | ConLS | ConR1 | sgRNA targeting *gfp* | AmpR | ColE1 | (17) |
| pMMK602 | ptac sgRNA | ConLS | ConR1 | sgRNA targeting *gfp* driven by ptac *lacO^-^* | AmpR | ColE1 | This study |
| pMMK603 | VioA sgRNA | ConLS | ConR1 | sgRNA targeting PL *vioA* driven by ptac *lacO^-^* | AmpR | ColE1 | This study |
| pMMK604 | macB sgRNA | ConLS | ConR1 | sgRNA targeting PL *macB* driven by ptac *lacO^-^* | AmpR | ColE1 | This study |
| pMMK809 | Stage 1 assembly | 1 | 5 | pBTK402-PA3-*NLuc*-T7 | KanR | RSF1010 | (17) |
| pMMK810 | Stage 1 assembly | 1 | 5 | pBTK402-CP25-*NLuc*-T7 | KanR | RSF1010 | (17) |
| pMMK811 | Stage 1 assembly | 1 | 5 | pBTK402-Ptac-*NLuc*-T7 | KanR | RSF1010 | This study |
| pMMK812 | Stage 1 assembly | 1 | 5 | pBTK402-macBp-*NLuc*-T7 | KanR | RSF1010 | This study |
| pMMK813 | Stage 1 assembly | 1 | 5 | pBTK402-macSp-*NLuc*-T7 | KanR | RSF1010 | This study |
| pMMK814 | Stage 1 assembly | 1 | 5 | pBTK402-CP25-*gfp*-T7 | KanR | RSF1010 | (17) |
| pMMK815 | Stage 1 assembly | N/A | N/A | pCRISPRi-*dCas9*-*bla*-Ptac-*gfp* | KanR/AmpR | RSF1010 | This study |
| pMMK816 | Stage 1 assembly | N/A | N/A | pCRISPRi-*dCas9*-*bla*-Ptac-*vioA* | KanR/AmpR | RSF1010 | This study |
| pMMK817 | Stage 1 assembly | N/A | N/A | pCRISPRi-*dCas9*-*bla*-Ptac-*macB* | KanR/AmpR | RSF1010 | This study |

**Table S4. List of Primers used in this study.**

| **Primer** | **Sequence** |
| --- | --- |
| p107_bbamp_F | TATGTGAGACCAGACCAATAAAAA |
| p107_bbamp_R | CGTTTGAGACCGACTACGGTTA |
| macb_seq_f | ATGAGCCGAGAATTATCCTTGAG |
| sheath_seq_f | CATGGCGTCATAGCAGTACA |
| ptac_gbsn_F2 | TAACCGTAGTCGGTCTCAAACGGCACTCCCGTTCTGGATAAT |
| ptac_gbsn_R2 | TTTTTATTGGTCTGGTCTCACATAGGGACAACTCCAGTGAAAAG |
| pBTK615_ptac_macB1_sgRNA_F | TCGGCTCGTATAATGTGTGGAAGCTCGGGGATCTGTCGTG |
| pBTK615_ptac_macB1_sgRNA_R | TTTTAACTTGCTATTTCTAGCTCTAAAACCACGACAGATCCCCGAGCTT |
| pBTK107_macB_promoter_gbsn_F1 | GATAACCGTAGTCGGTCTCAAACGGAAGTTTCTGCGGTGCTTTT |
| pBTK107_macB_promoter_gbsn_R1 | TTTTTATTGGTCTGGTCTCACATAAGATTACCTTATTAATGTTATTAATGAGCAT |
| pBTK107_sheath_promoter_gbsn_F1 | GATAACCGTAGTCGGTCTCAAACGACACCGACTTTACCCTATCTCG |
| pBTK107_sheath_promoter_gbsn_R1 | TTTTTATTGGTCTGGTCTCACATAGTTTTTCCTTACGTTGATAATTACATTC |
| pBTK107_CP25_F | TGAGGGGGCTGGTATAATCA |
| gRNA_VioA5_F | CACATATTTATGTTCATAAACTCGAAG |
| pBTK615_ptac_seqF1 | ACAGACACTGCGACAACGTG |
| pBTK615_gRNA_GFP | CGTCTAATTCCACGAGGATTG |
| p615_ptac_gRNA_VioA5_F2 | TCGGCTCGTATAATGTGTGGTTTATGTTCATAAACTCGAA |
| p615_ptac_gRNA_VioA5_R2 | TTTTAACTTGCTATTTCTAGCTCTAAAACTTCGAGTTTATGAACATAAA |
| p615_ptac_vector_amplification_R | CCACACATTATACGAGCCGA |
| p615_vector_amplification_F | GTTTTAGAGCTAGAAATAGCAAGTTAAAA |
| p615-ptac_F2 | CAATTAATCATCGGCTCGTATAATGTGTGGCGTCTAATTCCACGAGGATTG |
| p615-ptac_R2 | TACGAGCCGATGATTAATTGTCAACAGCTCTTCAGTGAGACGGTATTGCG |
| 61A2_Kan_intF2 | CTGCCTCGGTGAGTTTTCTC |
| 61C9_p614-bla_R1 | CTTTTCTACGGGGTCTGACGCGTCTCATGCTCCTCAGTGGAACGAAAACTCACG |
| 61D1_p614-bla_F1 | GTGAACACTCTCCCGGCTGAAATCTGCTCGTCAGTGGTG |
| 61D2_p614-bsmBI_R1 | GACGCGTCTCATGCTCCT |
| 61D2_p614-bsmBI_F1 | CTCCCGGCTGAAATCTGC |
| 62B5_pBTK107_seq_F | TGGATAACCGTAGTCGGTCTC |
| 62B6_pBTK107_seq_R | GGATTTGTTCAGAACGCTCGGTT |

**SUPPLEMENTAL FIGURES**

**
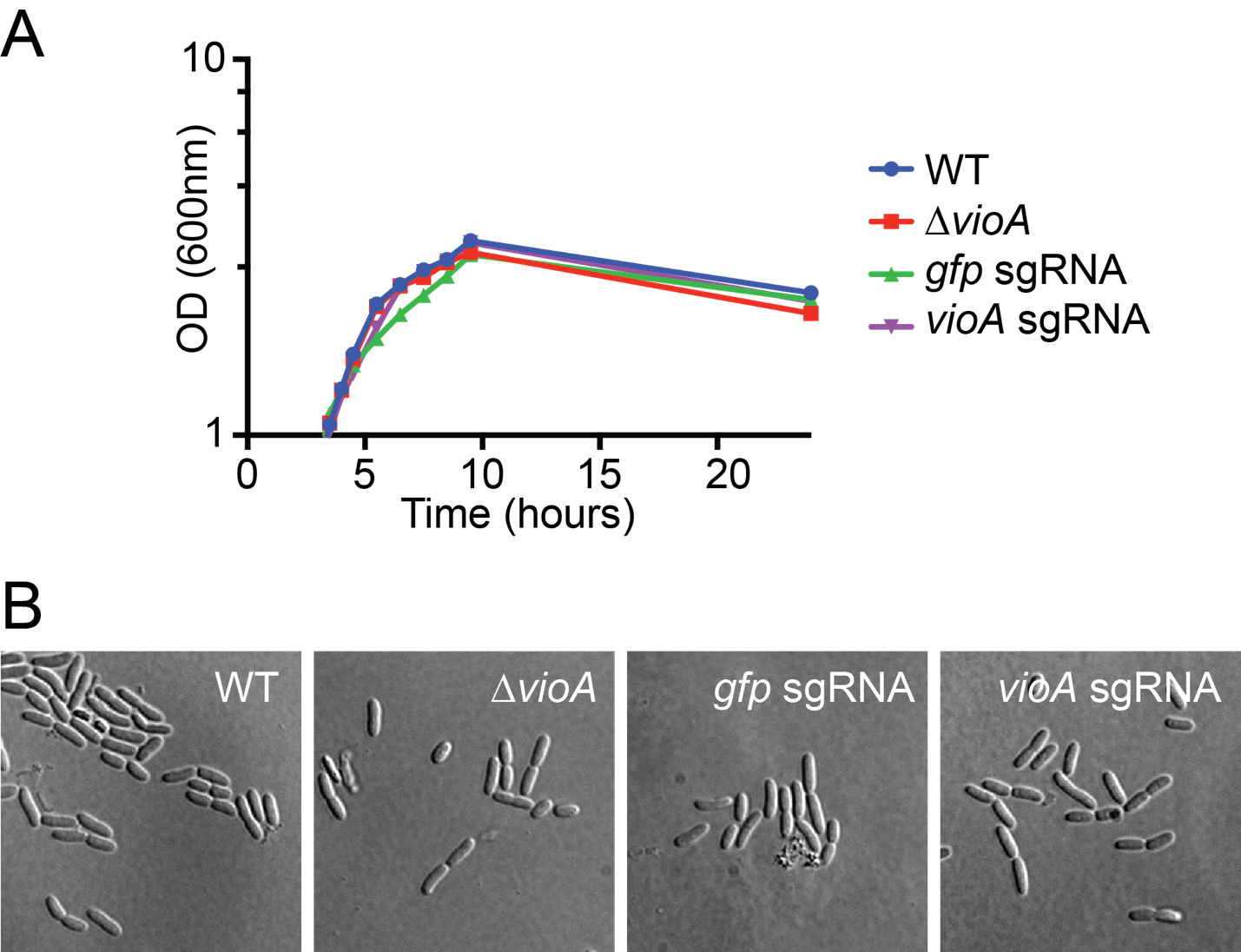
**

**Figure S1. MMK CRISPRi plasmids do not affect *P. luteoviolacea* growth or cell morphology.** (A) Growth curve and (B) DIC micrographs of *P. luteoviolacea* wild type (WT), ∆*vioA* or *P. luteoviolacea* strains with CRISPRi plasmids targeting *gfp* and *vioA*. Optical density (OD) measurements were taken at 600 nm wavelength and graphed on a Log10 scale.

**SUPPLEMENTAL MOVIES**

**Movie S1. Ingestion of *P. luteoviolacea* by *Hydroides* juvenile.** Merged fluorescence and DIC timelapse movie of *Hydroides elegans* juveniles imaged 24 hours after the competent larvae were exposed to inductive biofilms of *P. luteoviolacea* containing a plasmid with CP25-*gfp*. Bacteria can be seen collecting in the pharynx, then moving in a peristaltic fashion toward the gut Scale bar is 100 µm.
